# Chronic Abdominal Pain in Children and Adolescents: Parental Threat Perception Plays a Major Role in Seeking Medical Consultations

**DOI:** 10.1155/2016/3183562

**Published:** 2016-11-24

**Authors:** Claudia Calvano, Petra Warschburger

**Affiliations:** Department Psychology, Counselling Psychology, University of Potsdam, Potsdam, Germany

## Abstract

*Background*. Pain symptoms, associated impairment, and parental perception of threat are reported to be predictors of health care utilization (HCU) in childhood chronic abdominal pain (CAP). However, mediating variables and their interrelations have not yet been systematically studied.* Objectives*. This study aims to identify mediating pathways of influence between child's abdominal pain and the number of pain-related medical visits.* Methods*. In a multicenter study, we recruited* N* = 151 parent-child dyads with children aged 6–17 years suffering from CAP. A composite measure of pain symptoms was defined as predictor and the number of pain-related medical visits as outcome variable. This relation was analyzed by serial mediation, including child- and parent-reported impairment and parental threat perception as mediators.* Results*. Only parental threat perception significantly linked child's pain symptoms to the number of medical visits. Measures of impairment did not have a significant effect.* Conclusions*. Parental pain-related threat perception is strongly related to health care seeking in childhood CAP. Addressing threat perception might be a fruitful parent-centered approach in clinical practice.

## 1. Introduction

Chronic abdominal pain (CAP) is one of the most frequent bodily complaints in childhood and adolescence [1, 2] and is associated with a high psychosocial burden, poor functioning, and low health-related quality of life [3, 4]. Furthermore, CAP represents the most common reason for consultations with pediatric gastroenterologists [5], and children with the condition show higher health care utilization than children suffering from headaches or other bodily symptoms [6]. Increased utilization of health care not only poses an economic burden due to repeated diagnostic procedures [7] and referral to higher-level medical services [8] but may also negatively influence the prognosis of child pain [9, 10].

Therefore, research in this area has concentrated on identifying predictors of pediatric health care utilization (HCU). Both the level of pain symptoms and the degree of pain-related impairment in everyday functioning are positively correlated with the number of pediatric visits [2, 11–16]. Studies taking both of these variables into account often reported a greater impact from the level of impairment than from pain per se [2, 17]. However, the assessment of pain-related impairment varied in terms of the source of information, mostly depending on the age of the sample. Many studies including a broad age range were unable to differentiate between child- and parent-reported impairment, as assessment depended on the child's age, with parent report used for younger children and self-report used for children aged 11 or older [14]. Moreover, in adolescent samples, some studies included only self-report (e.g., [17]) while others relied solely on parent report [18]. According to results by Toliver-Sokol et al. [15] from an adolescent chronic pain sample, only the parent report on activity restrictions, and not the adolescent self-report, was directly related to HCU. Therefore, the first research question of this study was to analyze whose perception of pain-related impairment in everyday life, the one of the child or the one of the parent, might better explain health care seeking.

The second research question concerns the influence of parental beliefs regarding child's abdominal pain on health care seeking. Parental factors play an important role for HCU in childhood pain which was highlighted by several studies: Levy and colleagues [13] identified maternal psychological distress, that is, increased scores for anxiety, depression, or somatization, as strongest predictor for medical consultations, beyond child-reported pain. Taking also into account the study by Venepalli and colleagues [16], one can conclude that not only mother's own psychological strain [13] but also that maternal fear specifically regarding child's pain and long-term impairment influences HCU [16]. These beliefs relating to the significance of child's chronic pain are captured by the construct of parental threat perception regarding child's pain.

Evidence regarding parental threat perception in childhood chronic pain can be derived from various research contexts, that is, experimental, observational, and interventional studies. For instance, in experimental research, parental threat perception was induced by delivering threatening information regarding child's pain in the trials [19–21]. When parents are to believed that the pain imposes a high threat on the child, then not only did the parents expect higher pain levels for the child [19], but also parents were more attentive to the child [20, 21]. In studies assessing parental threat perception by questionnaire, it has been observed that increased levels of parental threat perception correspond with increased child pain intensity and a poorer adaptation profile [22]. Furthermore, Levy and colleagues showed that parental threat perception was significantly reduced after participation in a cognitive-behavioral intervention for functional abdominal pain [23, 24]. Due to variations in study design, the operationalization of threat perception differs across studies. Questionnaire assessment mostly operationalized threat perception as composite score of perceived pain symptom level (like intensity, duration, and frequency) and also the perceived seriousness and long-term impact of pain for child's health [22–24]. Regarding the latter dimension on pain as threat for child's long-term health, the context of health psychology research gives further evidence. In that field, threat perception is defined by two components: perceived vulnerability (or susceptibility) to illness and perceived severity (or seriousness) of symptoms [25]. This definition of threat perception has emerged as one key factor for the initiation of health-related behaviors [26]. Accordingly, parental threat perception seems to be a very promising construct to explain HCU in childhood pain as well. Up to now, in pediatric pain research, there is evidence that appraisals of the child's health status [11, 12] and child vulnerability for illness [27] significantly contribute to care seeking as well. However, the role of parental threat perception in childhood CAP for health care utilization has not been analyzed so far.

To date, studies have mainly examined direct effects on HCU, thus precluding statements about possible mediating variables. Moreover, most studies included either the child's or parents' view on impairment, thus impeding evidence on whose perception of impairment might be more relevant for HCU. To extend current knowledge on child and parent influences on HCU, this study applies a concurrent analysis of child and parent mediators of HCU in a sample suffering from chronic abdominal pain.

**Figure 1:**
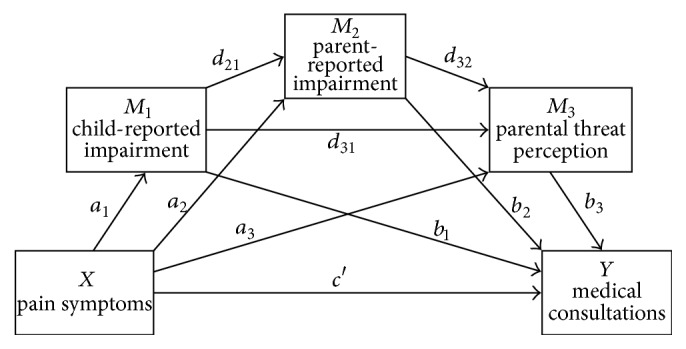
Conceptual model for serial mediation in the relation between child's pain symptoms and medical consultations, including coefficients for the direct pathways (*a*_*i*_ for pathways between predictor pain symptoms and each mediator *M*_*i*_; *b*_*i*_ for pathways between each mediator *M*_*i*_ and the outcome medical consultations; *d*_*ij*_ for serial pathways between the mediators; *c*′ for the direct effect between pain symptoms as predictor and medical consultations as outcome).

Based on current evidence [2, 13–15], we first assume a direct positive association between the child's pain symptoms and the number of pediatric visits. We aim to explain this relationship by including impairment and parental threat perception as mediators. In order to assess distinct influences, we include both child and parent reports of impairment in the model. Literature reviews on the interplay between child and parent factors in chronic pain (e.g., [28]) form the basis for our conceptual model. Assuming that parent's cognitive and behavioral reactions depend on child's pain experience and associated behavior, we postulate a serial mediation model as follows: child-reported impairment is defined as first mediating variable, explaining the link between pain symptoms and medical visits. As we hypothesize that the degree of parent's reported impairment is influenced by child's pain and impairment, this variable was defined as second mediator. Assuming that parental threat perception is based on the observation of the child's pain symptoms and influenced by the degree of impairment in everyday functioning, we postulate a serial mediation model for the interactive effects of these variables (Figure 1).

Using this model, specific effects of each mediator are analyzed separately as well as serially. We hypothesize (1) that impairment will mediate the relationship between pain reports and medical visits, (2) that when both child and parent report of impairment are taken into account, parent-reported impairment will exert the stronger influence, and (3) that threat perception will significantly explain additional variance.

Furthermore, we compare the strengths of the indirect effects by contrasting the coefficients. We hypothesize that the indirect pathways including threat perception as mediator (*M*; including *M*_3_, *M*_1_-*M*_3_, *M*_2_-*M*_3_, and *M*_1_-*M*_2_-*M*_3_) will exert a more pronounced influence than the pathways which only control for threat perception (*M*_2_, *M*_1_-*M*_2_).

## 2. Methods

### 2.1. Design

This cross-sectional study is part of a research project focusing on childhood CAP [4]. In 16 study centers, children suffering from chronic abdominal pain and their caregivers were consecutively recruited during visits to a pediatric gastroenterological outpatient clinic. Children underwent medical examination conducted by the pediatric gastroenterologist who also reported on their gastrointestinal diagnosis. Data for the research project were collected by questionnaires after parental informed consent and child assent. Over a 9-month period, 635 families with children aged 1–18 years presenting at the clinic were addressed, of which 34.8% complete parent-child dyads were returned. For this study, the inclusion criteria were as follows: age of child 6–17 years, presentation due to chronic abdominal pain, child and parent data available, informed parental consent, and child assent.

This study was approved by the Ethics Committee of the University of Potsdam (date 18/04/2011).

### 2.2. Participants

*N* = 151 parent-child dyads fulfilled inclusion criteria.  Table 1 summarizes the sociodemographic and utilization-related characteristics of the final sample. The medical diagnoses were as follows: 7.3% lactose intolerance, 27.8% fructose malabsorption, 7.9% both, 2.0% other carbohydrate malabsorption, 5.3% constipation, and 49.7% functional gastrointestinal disorder. We assessed possible differences between diagnostic groups (carbohydrate intolerance/constipation versus functional gastrointestinal disorder) on the main variables in our mediation model. Results are summarized in Table 2. As subgroups do not significantly differ on the measures, we combined the groups in one analysis.

### 2.3. Measures

*Pain Symptoms*. The children rated their pain intensity* (“How strong was your belly ache during the last 2 weeks?”)* and pain frequency* (“How often did you have belly ache during the last 2 weeks?”)* on a 6-point scale with verbal descriptions (from 0 “not at all” to 5 “very strong”/“daily”); the pain duration* (“How long does your belly ache usually last?”)* was rated on a 5-point scale with verbal descriptions (from 1 “less than one hour” to 5 “the whole day”). For the items on intensity and frequency, we used the faces derived from Wong-Baker Faces Pain Rating Scale (WBFPRS [29]) as additional answering format corresponding to the verbal descriptions of categories. The use of the WBFPRS served to take the broad age span into account and ease answering for the younger children in the sample. In a systematic review, this scale was reported as valid pain measure, highly accepted by children and therefore recommended for children from the age of 6 onwards [30]. Ratings of intensity, frequency, and duration were multiplied to form a comprehensive index of abdominal pain symptoms (cf. [31]).

*Pain-Related Impairment Child Self-Report*. We used the “interference” subscale of the Pain Experience Questionnaire [32], a validated measure for children and adolescents aged 7–18 years. The six items cover school and homework, leisure time, and family activities. The scale score correlates with pain measures and internalizing symptoms [32]. The original 7-point scale was changed into a 5-point Likert scale (from 1 “never” to 5 “always”) in order to correspond to the 5-point format of other scales in the child questionnaire [4]. We calculated a sum score with good internal consistency in our sample (Cronbach *α* = .89).

*Pain-Related Impairment Parent Report*. We used the parent version of the Pediatric Pain Disability Index (P-PDI [33]), which is a validated proxy report for adolescents aged 11–17 years. Parents rated the degree of their child's impairment in everyday life (12 items; e.g., reading, going to school, and sleeping) on a 5-point frequency scale (from “never” to “always”). We calculated a sum score, which yielded excellent internal consistency in our sample (Cronbach *α* = .92).

*Threat Perception*. In this study, threat perception was defined as a two-component construct including perceived vulnerability for long-term illness and perceived severity of abdominal pain for child's health. Based on established measures for threat perception in adult samples [25, 34], we adapted the wording of the two items for perceived vulnerability, respectively, perceived severity to the context of childhood CAP. Parents reported on their child's vulnerability (“How high do you rate the risk of long-term impairment for your child because of abdominal pain?”; cf. [35]) and on perceived severity of pain for child's health (“How severe do you rate the abdominal pain for the health of your child?”; cf. [36]). For each item, scaling ranged from 1 to 7, with anchors on 1 (not very likely/not severe), 4 (moderately likely/moderately severe), and 7 (very likely/very severe). Threat perception was computed as a mean score, with sufficient internal consistency (Cronbach *α* = .77).

*Health Care Utilization*. In line with the literature [13, 15], health care utilization was assessed using an open-response format based on parent report* (“In the last 6 months, how often did you visit a doctor because of your child's abdominal pain?”)*.

*Sociodemographic Factors*. Children's age and gender were collected based on parent report. Parents also reported their marital status, educational background, and provided descriptive data on the child's health care use.

### 2.4. Data Analysis

The final dataset was analyzed using SPSS 22 for Windows. The serial mediation analysis based on OLS regression was conducted using the PROCESS macro for SPSS by Hayes [37, 38]. Missing values in latent variables were substituted with the EM algorithm when the proportion of missing data per variable did not exceed 5% [39], which applied for the predictor and all the three mediator variables in the conceptual model. For the outcome variable of medical consultations due to abdominal pain, there were missing data in 10 cases (6.6%), exceeding the threshold for substitution set in this study. Therefore, these missing values were not replaced by EM imputation. As the PROCESS macro does not allow for missing data, for mediation analysis and bootstrapping, these 10 cases were excluded from analysis; in addition, 3 cases had to be excluded due to indistinct reports on HCU (no visits in the last 6 months) which results in total *N* = 138 for the serial mediation analysis.

Our model is based on a direct effect of pain symptoms on medical consultations (*c*′). According to our hypotheses, impairment based on child self-report was defined as first mediator (*M*_1_), followed by parent report of impairment (*M*_2_) and parental threat perception (*M*_3_), resulting in three specific indirect effects, through *M*_1_ (*a*_1_ *b*_1_), *M*_2_ (*a*_2_ *b*_2_) or *M*_3_ (*a*_3_ *b*_3_). In addition, effects in sequence were analyzed, that, is over the sequence *M*_1_-*M*_2_ (*a*_1_ *d*_21_ *b*_2_), *M*_1_-*M*_3_ (*a*_1_ *d*_31_ *b*_3_), and *M*_2_-*M*_3_ (*a*_2_ *d*_32_ *b*_3_) or over all mediators in serial: *M*_1_-*M*_2_-*M*_3_ (*a*_1_ *d*_21_ *d*_32_ *b*_3_). Notably, in each subanalysis, the remaining mediator(s) were statistically controlled for. Pairwise comparisons of coefficients (contrasts) examined the strength of effects, that is, whether and how indirect effects differed from each other. Statistical significance was based on 95% bias-corrected and accelerated confidence intervals for the regression coefficient (95% BCa CI) [40], using 10.000 bootstrap samples.

## 3. Results

Due to missing cases in the outcome “medical consultations,” the sample size in statistical analyses and hypotheses testing refers to *N* = 138. Bivariate correlations and descriptive statistics for the variables in the model are summarized in Table 3. In the first step, we checked correlations with main sociodemographic measures (age, gender, and socioeconomic status) to identify possible covariates in the model. The number of medical consultations was significantly correlated with the child's age (*r* = 0.216,* p* = 0.011), but not with the parents' age (*r* = 0.114,* p* = 0.184). As the child's age was also significantly correlated with pain symptoms (*r* = 0.224,* p* = 0.006), parent-reported impairment (*r* = 0.279,* p* < 0.001), and threat perception (*r* = 0.243,* p* = 0.003), we included the child's age as covariate in the analysis. There were no significant correlations with parent gender or socioeconomic status.

The results with respect to the single pathways in the model (see Figure 1) are summarized in Table 4. In line with the conceptual model, we found a significant association between the child's pain and number of medical consultations (total effect* B* = 0.621, SE = 0.287,* p* = 0.032, 95% BCa CI [0.054, 1.188]), explaining 7.8% of the variance (*F*(2,135) = 5.752,* p* = 0.004). When the mediators were included in the analysis, this coefficient was no longer statistically significant (direct effect *c*′;* B* = 0.132, SE = 0.326,* p* = 0.687, 95% BCa CI [−0.513,0.776]).

**Figure 2:**
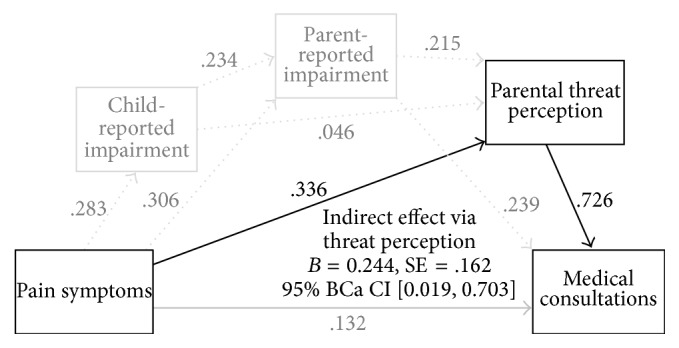
Final mediation model for the relation between pain symptoms and medical consultations with coefficients for the direct pathways (*N* = 138). Pathways marked in grey indicate the nonsignificant effects.

The results for the specific and serial mediation effects are summarized in Table 5. Summing up all indirect effects yielded a significant result (total indirect effect,* B* = 0.489, SE = 0.226, 95% BCa CI [0.125,1.008]). In line with hypothesis 1, self-report of impairment did not have a significant specific indirect effect when parental measures were controlled for. In contrast to hypothesis 2, proxy report of impairment also did not have a specific effect. Threat perception proved to be a significant mediator, which is in line with hypothesis 3. For serial effects, no pathway yielded statistical significance. As we only had one statistically significant mediation effect, contrasts were suspended with. The final model is depicted in Figure 2.

## 4. Discussion

We analyzed influences on children's medical consultations in an outpatient, secondary care sample of children and adolescents suffering from CAP. By integrating self- and parent-reported impairment and parental threat perception simultaneously, this analysis extended the current knowledge on predictors of pediatric health care seeking in three ways. First, the concurrent analysis of both child- and parent-reported impairment provided insight into the relevance of the parents' view independent of the child's age; second, the concurrent analysis enabled conclusions to be drawn about the influence of parental threat perception beyond impairment; and third analyzing parents' threat perception provided first insight into its importance for medical utilization in childhood CAP.

In line with the literature, pain symptoms were strongly and positively correlated with the number of medical consultations [11, 14, 15]. In agreement with our hypothesis, the influence of child-reported impairment disappeared when parental measures were taken into account. This underlines that parents' view on impairment plays a major role in explaining pediatric consultations [12, 15, 16]. In addition, we found different contributions of perceived impairment and threat perception. Despite high coefficients on direct pathways in the model for both variables, only parental threat perception significantly linked pain symptoms and number of medical visits. This observation extends previous studies reporting that pain symptoms and related impairment are stable predictors of HCU [2, 6, 11, 14, 15] insofar as we now point to the dominant mediating role of parental threat perception for HCU.

Our study should be discussed in light of the results by Venepalli et al. [16], who identified that maternal fear of persistence and long-term impairment differentiated between consulting versus nonconsulting families. Focusing on consulting families, we have now identified threat perception, which comprises comparable worries, as the major influence for seeking medical visits. We also add to results by Connelly et al. [27], who identified parental perceptions of child vulnerability to illness as a mediating variable for HCU, by operationalizing threat perception as a very pain-specific measure, taking perceptions of vulnerability and severity of the child's pain into account. The role of parent's threat perception regarding child's pain is underlined in a variety of studies. While the results for this study identified threat perception as central mediator for medical health care seeking, the observations in an interventional study by Levy and colleagues deliver further evidence as well. Applying three common parent and child sessions based on social learning theory, the authors not only report that parental threat perception was significantly reduced after treatment [23, 24], but also identified reductions in parental threat perception as mediator, explaining the decrease in child's pain after treatment [41]. Some studies have examined threat perceptions in children as well. In an earlier study, Lipani and Walker [42] identified that child's threat appraisal is associated with maternal distress and impairment in family functioning. Walker and colleagues [22] reported a high correspondence between child- and parent-reported threat perceptions. Assuming that threat perceptions are significant beliefs for various outcomes in childhood pain and in order to gain a deeper understanding on family processes in chronic pain [28], future studies should explore correlates of threat perception on the family level as well. By including a broad age range as well as both self- and proxy reports of impairment, we aimed to clarify the relative influence of impairment on HCU. We hypothesized that, for pediatric health care seeking, parental perceptions play a major role, even for adolescents, who may be more autonomous in terms of medical visits. 27.2% of our sample was aged 13–17 years. Increasing child age was accompanied by increasing values on all variables except for self-reported impairment. Our results suggest that the parental perspective is the more influential source of information, probably acting through their perception of threat, in explaining HCU.

The results of this study have to be seen in the context of methodological strengths and limitations. According to recommendations for the assessment of pain [30, 43], our model assessed pain in child self-report. We used a retrospective cross-sectional recall by the children to cover the last 2 weeks. While a retrospective recall might be biased, a recent study in children and adolescents aged 8–18 years suffering irritable bowel syndrome did not find significant differences between retroversus prospective diary approaches for pain assessment [44]. Nonetheless, our retrospective approach of pain assessment has to be seen as limitation. In line with current reviews and recommendations on the interplay of child and parent factors [28, 45], we integrated both child- and parent-report of impairment into one mediation analysis. Furthermore, by analyzing impairment and parental threat perception concurrently in one analysis, we were able to analyze their distinct contribution for HCU. The sample covered a broad age range, and a possible confounding age effect was statistically controlled for. With regard to methodological aspects, the serial model implies causal links between the mediators. However, the cross-sectional design precludes causal conclusions, as the interrelations between variables in the model might be epiphenomenal, due to one common cause, or even causal in nature [37]. Future research should longitudinally analyze causal agents for parental threat perception and HCU. While the application of an OLS-regression-based analysis and underlying *t*-distribution ensures more accurate *p* values for the path coefficients in smaller samples like in this study [37], research with larger sample sizes would enable path analysis configurations and estimations of latent variable models.

The generalizability of the results might be limited due to the preselected clinical sample in secondary care and the fact that the sample was well-educated, with over 80% of parents having a medium to high educational background. In addition, a larger sample size across various diagnostic subgroups might allow for intergroup differences in the meditational model and distinct pathways. In this study, separate analyses were not conducted as the model lacked statistical power. Reliance on parent report may lead to shared method variance accounting for relations among threat perception and HCU; however, by analyzing pain and impairment based on child report, we aimed to reduce this limitation.

Our findings allow several implications for future research and clinical practice. Based on our results, parents' threat perception might constitute one approach to health care utilization, underlining the central role parents play in frequent pediatric visits. According to parents' individual threat evaluation, doctor visits may be adequate reactions to a child's chronic abdominal pain. However, the potential course from an adaptive behavior to highly increased medical utilization as maladaptive coping needs to be studied in future research. Longitudinal analysis of outcomes in high-utilizing families is warranted. Threat perception seems to only partly reflect child's pain and related impairment, as these variables explained 24.7% of variance of threat perception in our sample. In clinical practice, a thorough examination of what actually drives threat perception should be undertaken. Not only are increased psychological symptoms highly prevalent among parents of children suffering from CAP [46, 47], maternal psychological well-being was identified as distinct characteristic in consulting families as well [13]. Therefore, one further can assume that parental symptoms of anxiety or health anxiety might influence their threat perception concerning child's abdominal pain and also pediatric HCU as well. This relationship needs to be studied in future research.

Our results suggest that addressing parental threat perception might be a fruitful component in parent-centered counseling in the pediatric setting. Accompanied by skilled health care communication [48], delivering information about the natural course and prognosis of abdominal pain can specifically target the threat perception. This might help to foster a biopsychosocial understanding of illness and enable effective coping and adjustment for both child and parent. It has been shown that a biopsychosocial conceptual model of CAP positively influences the course of CAP [49] and might reduce excessive HCU as well [9]. Our results stress that we should not only aim to modify behavioral responses to the child's pain complaints, but should also target parents' pain-specific appraisals.

## 5. Conclusions

As chronic abdominal pain is the most common reason for consultations of pediatric gastroenterologists, this study aimed to identify significant pathways to pain-related medical seeking in secondary care. When parental threat perception is taken into account, the degree of pain-related impairment in functioning did not significantly contribute to health care seeking, neither in child, nor in parent report. Only parent's threat perception significantly linked child's pain to the number of medical visits. Therefore, the parental influence is mainly acting through their subjective perception of threat, less through their perception of child's impairment. Targeting parental threat perception in the pediatric setting might be a promising way to foster a biopsychosocial model of chronic pain and that way to reduce increased health care use.

## Figures and Tables

**Table 1:** Sample characteristics (*N* = 151 parent-child dyads).

Variable	
*Child age*	
M	10.95
(SD)	(2.64)
Range	6–17
*Parent age*	
M	41.92
(SD)	(5.91)
Range	27–56
*Gender child*	
% (female/male)	64.9/35.1
*Gender parent*	
% (female/male)	85.4/14.6
*Single parent*	
% (yes/no)	17.2/82.8
*Education parent*	
% (low/medium/high)	18.5/41.1/40.4
*Duration since onset*	
% (<3/4–11/≥12 mo.)	19.2/30.5/50.3
*Total duration of medical care (months)*	
M	17.40
(SD)	(27.22)
Range	0–132
*Number of different physicians seen within last 6 months*	
M	3.98
(SD)	(3.36)
Range	1–25
*Low versus high utilizer*	
% (low/high)	62.9/37.1

*Note*. High utilizer ≥ 4 visits in last 6 months; education was coded as low (no school-leaving qualifications or special school), medium (secondary school), and high (diploma or university degree).

**Table 2:** Descriptive data and results of group comparisons on measures included in the meditation model (*N* = 151).

	Carbohydrate intolerance and constipation (CC) *N* = 76		Functional gastrointestinal disorder (FGID) *N* = 75		*p*
	M (SD)	Range	M (SD)	Range	
Medical consultations^1^	3.80 (3.40)	1–25	4.15 (3.33)	1–20	0.544
Pain symptoms	29.42 (30.98)	0–125	31.74 (28.88)	0–125	0.636
Impairment self-report	49.60 (29.12)	0–100	56.33 (26.17)	0–100	0.137
Impairment parent-report	35.89 (20.57)	0–100	36.14 (19.13)	0–79.17	0.937
Threat perception	47.22 (23.10)	0–100	47.83 (22.85)	0–100	0.871

*Note*. Except for health care utilization and pain symptoms, scale scores were transformed to a range from 0 to 100. ^1^Medical consultations refer to the number of consultations due to the child's abdominal pain in the last 6 months (due to missing data*N* = 68 for HCU in the CC group; *N* = 70 for HCU in the FGID group).

**Table 3:** Descriptive statistics and bivariate correlations (*N* = 151).

Variable		2	3	4	5	M (SD)	Range
1	Medical consultations^1^	0.228^*∗∗*^	0.201^*∗*^	0.246^*∗∗*^	0.313^*∗∗∗*^	3.98 (3.36)	1–25
2	Pain symptoms	—	0.302^*∗∗∗*^	0.434^*∗∗∗*^	0.457^*∗∗∗*^	30.57 (29.87)	0–125
3	Impairment self-report	—	—	0.376^*∗∗∗*^	0.256^*∗∗*^	52.94 (27.81)	0–100
4	Impairment parent-report	—	—	—	0.391^*∗∗*^	36.01 (19.80)	0–100
5	Threat perception	—		—	—	47.53 (22.90)	0–100

*Note*. Except for health care utilization and pain symptoms, scale scores were transformed to a range from 0 to 100. ^*∗*^ *p* < 0.05, ^*∗∗*^ *p* < 0.01, and ^*∗∗∗*^ *p* < 0.000. ^1^Medical consultations refer to the number of consultations due to the child's abdominal pain in the last 6 months (*N* = 138 due to missing data on medical consultations).

**Table 4:** Results for the pathways in the serial mediation model for the prediction of medical consultations (*N* = 138).

Antecedent	Consequent															
	*M*_1_ impairment self-report				*M*_2_ impairment proxy report				*M*_3_ parental threat perception				*Y* medical consultations			
		Coeff.	SE	*p*		Coeff.	SE	*p*		Coeff.	SE	*p*		Coeff.	SE	*p*
*X* (pain symptoms)	*a*_1_	0.283	0.086	0.001	*a*_2_	0.306	0.078	0.000	*a*_3_	0.336	0.083	0.000	*c*′	0.132	0.326	0.687
*M*_1_ (impairment self-report)		—	—	—	*d*_21_	0.234	0.075	0.002	*d*_31_	0.046	0.079	0.559	*b*_1_	0.316	0.291	0.280
*M*_2_ (impairment proxy report)		—	—	—		—	—	—	*d*_32_	0.215	0.087	0.015	*b*_2_	0.239	0.329	0.469
*M*_3_ (parental threat perception)		—	—	—		—	—	—		—	—	—	*b*_3_	0.726	0.320	0.025
Covariate (child age)		0.030	0.032	0.352		0.076	0.029	0.009		0.030	0.030	0.310		0.149	0.109	0.176
Constant	*i*_*M*1_	−0.323	0.362	0.374	*i*_*M*2_	−0.784	0.319	0.015	*i*_*M*3_	−0.328	0.329	0.320	*i*_*Y*_	2.331	1.217	0.058
		*R* ^2^ = .095				*R* ^2^ = .282				*R* ^2^ = .274				*R* ^2^ = .138		
		*F*(2,135) = 7.082				*F*(3,134) = 17.509				*F*(4,133) = 12.515				*F*(5,132) = 4.231		
		*p* = 0.001				*p* < 0.001				*p* < 0.001				*p* = 0.001		

*Note.* Coeff. = unstandardized regression coefficient *B*. Predictor variables were *z*-standardized. Child age was included as covariate.

**Table 5:** Results of serial mediation analysis for specific and serial indirect effects (*N* = 138).

	Hypothesis	Mediator(s)	Indirect effect	*B*	SE	95% BCa CI	
						Lower	Upper
Specific effects	1	imp_self_	*a*_1_ *b*_1_	0.089	0.080	−0.038	0.287
	2	imp_proxy_	*a*_2_ *b*_2_	0.073	0.114	−0.121	0.340
	3	Threat perception	*a*_3_ *b*_3_	0.244	0.162	0.019	0.703

Serial effects	4	imp_self_ - imp_proxy_	*a*_1_ *d*_21_ *b*_2_	0.016	0.028	−0.022	0.097
	5	imp_self_ - threat perception	*a*_1_ *d*_31_ *b*_3_	0.010	0.026	−0.017	0.112
	6	imp_proxy_ - threat perception	*a*_1_ *d*_32_ *b*_3_	0.048	0.050	−0.001	0.210
	7	imp_self_ - imp_proxy_ - threat perception	*a*_1_ *d*_21_ *d*_32_ *b*_3_	0.010	0.014	0.000	0.066

*Note*. imp_self_ = impairment based on self-report; imp_proxy_ = impairment based on parent report.
